# Phenotype of NK Cells Determined on the Basis of Selected Immunological Parameters in Children Treated due to Acute Lymphoblastic Leukemia

**DOI:** 10.1097/MD.0000000000002369

**Published:** 2015-12-31

**Authors:** Sylwia Koltan, Robert Debski, Andrzej Koltan, Elzbieta Grzesk, Barbara Tejza, Andrzej Eljaszewicz, Lidia Gackowska, Malgorzata Kubicka, Beata Kolodziej, Beata Kurylo-Rafinska, Izabela Kubiszewska, Malgorzata Wiese, Milena Januszewska, Jacek Michalkiewicz, Mariusz Wysocki, Jan Styczynski, Grzegorz Grzesk

**Affiliations:** From the Departments of Pediatrics, Hematology and Oncology; and Immunology (SK, RD, AK, EG, BT, AE, LG, MK, BK, BK-R, IK, M Wiese, MJ, JM, M Wysocki, JS), Collegium Medicum in Bydgoszcz, Bydgoszcz, Poland, and Department of Pharmacology and Therapeutics, Faculty of Medicine, Collegium Medicum in Bydgoszcz, Nicolaus Copernicus University in Torun, Torun, Poland (GG).

## Abstract

Acute lymphoblastic leukemia (ALL) is the most frequent pediatric malignancy. The chemotherapy for ALL is associated with a profound secondary immune deficiency.

We evaluated the number and phenotype of natural killer (NK) cells at diagnosis, after the intensive chemotherapy and following the completion of the entire treatment for patients with ALL. The fraction, absolute number, and percentage of NK cells expressing interferon-γ were determined in full blood samples. The fraction of NK cells expressing CD158a, CD158b, perforin, A, B, and K granzymes was examined in isolated NK cells.

We have shown that patients assessed at ALL diagnosis showed significantly lower values of the fraction of NK cells and percentage of NK cells with the granzyme A expression. Additionally, the absolute number of NK cells, the expression of CD158a, CD158b, perforin, and granzyme A were significantly lower in patients who completed intensive chemotherapy. Also, there was a significantly higher fraction of NK cells expressing granzyme K in patients who completed the therapy.

Abnormalities of NK cells were found at all stages of the treatment; however, the most pronounced changes were found at the end of intensive chemotherapy.

## INTRODUCTION

Acute lymphoblastic leukemia (ALL) is the most frequent childhood malignancy.^1,2^ Its etiology is not fully understood in the vast majority of patients; nevertheless, curability reaches 80% to 85%.^3,4^ Standard chemotherapy, constituting the base of the treatment, is associated with severe secondary immune deficiency leading to serious infectious complications.^5,6^

Natural killer (NK) cells are the components of the innate immunity, representing the first line of defense against infections and neoplastic transformation. In order to execute their biological functions, they have to “learn” to distinguish between own healthy cells and those altered in the course of infection or proliferative process. Inhibiting and activating receptors belong to the main components of this “recognition.” Killer immunoglobulin-like receptors (KIRs) play an important role among NK receptors.^6–13^

NKs execute their effector function using 2 mechanisms. First of all, approximately 95% of the peripheral blood cells, expressing CD56dim+ CD16+, show cytotoxicity associated with perforin and granzymes, among which A, B, and K seem the most important. Secondly, the remaining 5% of NKs show the expression of CD56bright+ CD16− and act through the release of cytokines, with interferon-γ (INF-γ) being the most important in this case.^14–18^

The information on the role of NKs in children suffering from ALL and their disorders at various stages of treatment is limited; the same is true about the data on the influence on the risk of severe infectious complications. Therefore, we analyzed the count and phenotype of NK cells on the basis of selected immunological parameters determined at diagnosis, as well as after completing intense therapy and comprehensive treatment of standard- and intermediate-risk ALL according to the ALL IC-BFM 2002 protocol for children and young adults.

### Patients

The study included 49 individuals between 1.2 and 19.2 years of age (median [Me] 5.3 years) at diagnosis of standard- and intermediate-risk ALL. There was a slight predominance of girls (53.1%) in this group (Table 1). The participants were divided into 3 groups in order to determine the immunological parameters characterizing the phenotype of NK cells:

- 21 children between 1.1 and 17 years of age (Me 4.7 years), who were examined at diagnosis of malignancy;
- 22 patients who completed the intense phase of the chemotherapy; their age at diagnosis and examination ranged between 1.3 and 17 years (Me 4.9 years) and 2.1 and 18 years (Me 5.8 years), respectively;
- 23 patients who completed the entire comprehensive oncological therapy, their age at diagnosis and examination ranged between 1.6 and 19.2 years (Me 6.6 years), and 4 and 21.3 years (Me 8.9 years), respectively (Table 1).

**TABLE 1:**
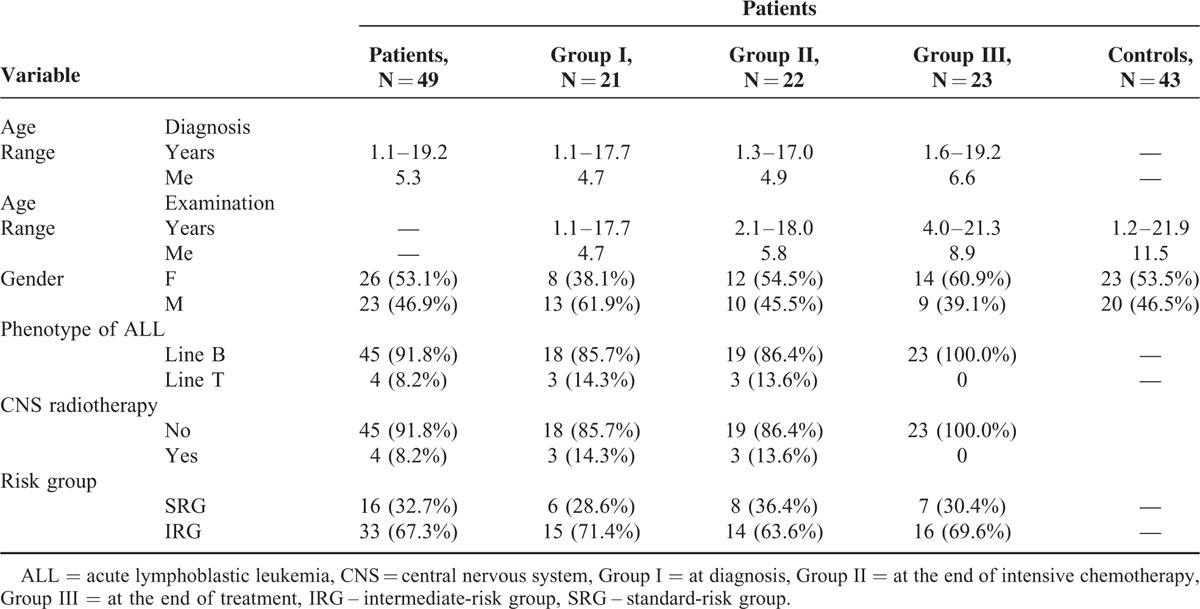
Characteristic of Patients and Controls

**FIGURE 1:**
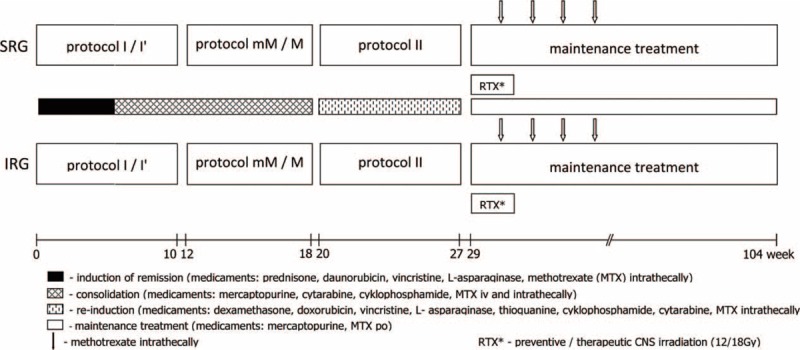
Acute lymphoblastic leukemia IC-BFM 2002 treatment protocol.

All patients were treated in accordance with the ALL IC-BFM 2002 protocol for standard- and intermediate-risk groups (Fig. 1).

The control group was comprised of 43 individuals’ ages between 1.2 and 21.9 years (Me 11.5 years), girls constituted 53.5% and boys 46.5% (Table 1).

### NK Isolation and Flow Cytometric Analysis

The phenotype of NKs in patients was analyzed at 3 time points: at diagnosis (group I), about 2 weeks after completing the intense phase of ALL IC-BFM 2002 protocol-mandated chemotherapy, immediately prior to remission maintenance therapy (group II), and 2 weeks after completing the maintenance therapy (group III). Full blood samples were examined in all patients and controls in order to analyze the lymphocyte subpopulations and the intracellular expression of IFN-γ; the remaining analyses involved isolated NK cells.

The lymphocyte subpopulations were analyzed with the panel of the following antibodies: CD2(FITC)/CD19(RPE), CD3(RPE-Cy5)/CD4(FITC)/CD8(RPE) (DAKO, Glostrup, Denmark), and CD3(FITC)/CD16+56+(PE) (Becton Dickinson—BD, Franklin Lakes, NJ). Cytomix FC500 cytometer (Beckman-Coulter, Fullerton, CA) was used for cytometric analysis.

For the evaluation of intracellular expression of IFN-γ in NKs of the patients heparinized whole blood was lysed using PharmLyse buffer (BD Biosciences, Warszawa, Poland ). Thereafter, cells were stained with the following panel of fluorochrome-conjugated monoclonal Abs directed against cell surface markers: CD56(PE)/CD3(PerCP) all from BD Bioscience. Subsequently, cells were fixed and permeabilized using Cytofix/Cytoperm kit (BD Bioscience) and stained using murine fluorochrome conjugated monoclonal Abs directed against human IFN-γ from Santa Cruz Biotechnology (Santa Cruz, CA). Isotype control was used for every staining using the isotype of antibodies included in the staining panel. Flow cytometric data were acquired on FACScam flow cytometB (BD bioscience) and analyses with the use of FlowJo 7.6.5 software (Tree Star, Ashland, OR). Gates were set to include CD3-lymphocytes. Thereafter, NKs were defined by the expression of CD56. The percentage of IFN-γ producing NKs was determined by using isotype control.

NK cells were isolated by means of negative selection with Super-MACS separator for magnetic isolation and NK Cell Isolation Kit (Miltenyi Biotec; Bergisch Gladbach, Germany). The content of isolated NK cells was determined using Cytomix FC500 cytometer (Beckman-Coulter).

### Isolation of NK Cells

**FIGURE 2:**
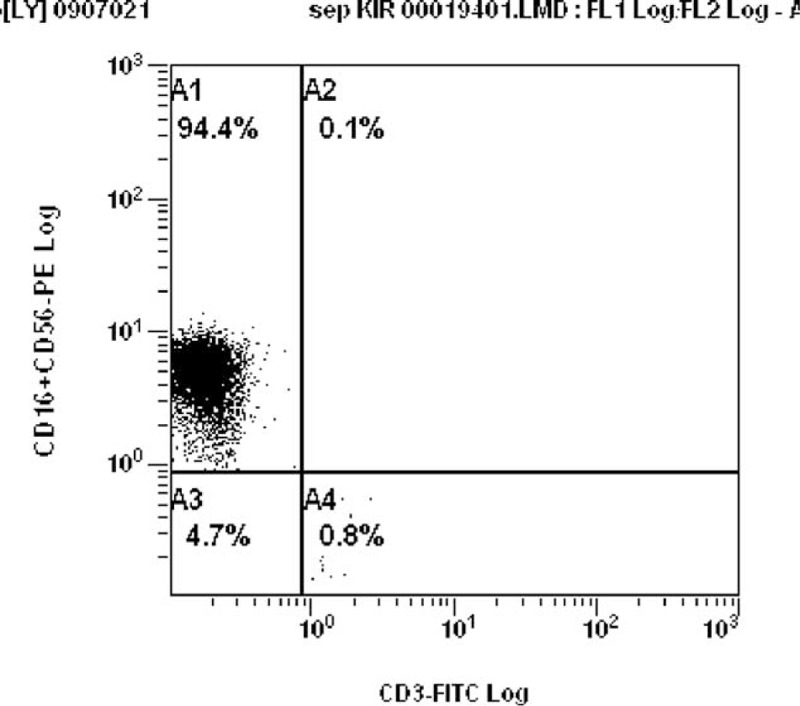
Quality of NK cells purification process. Purity isolation was evaluated using a panel of monoclonal antibodies CD16 + CD56-PE and CD3-FITC. NK cells are in a pool of cells expressing the CD16 + CD56 and the absence of expression of CD3. NK = natural killer.

The isolation of NK cells was performed on the basis of negative selection using a magnetic separator for magnetic isolation SuperMACS and a set for NK Cell Isolation Kit from Miltenyi Biotec, strictly according to the attached instructions. Kit for the isolation is a “cocktail” of monoclonal Abs against antigens having its expression in all cells, except NK cells. These antibodies are combined with magnetized micro-beads. The mixture of cells is located in small columns for the separation of LS Miltenyi Biotec. During the isolation process in SuperMACS separator cells combined with antibodies conjugated to the micro-magnets are removed from the mixture and remain only unmarked NK cells. Purity isolation was evaluated using a panel of monoclonal antibodies CD16+ CD56-PE and CD3-FITC. NK cells are in a pool of cells expressing the CD16+ CD56 and the absence of expression of CD3. In the diagram, they represent 94.4% of all cells (Fig. 2).

The expression of KIR on isolated NK cells was analyzed with the following monoclonal Abs: CD158a-FITC (BD Pharmingen, San Diego, CA), binding to 2DL1 and 2DS1 receptors, and CD158b-PE (BD Pharmingen), binding to 2DL2 and 2DL3 receptors. Cytometric reading with Cytomix FC500 cytometer (Beckman-Coulter) took place immediately after isolation and labeling of NK cells.

The expression of perforin and granzymes A, B, and K was determined in isolated NK cells subjected to permeabilization with Cytofix/Cytoperm kit (BD). The cells were labeled with the following murine fluorochrome conjugated monoclonal Abs: antiperforin-PE, antigranzyme A-PE from BD (BD Bioscience), antigranzyme B-PE (Invitrogen, Carlsbad, CA), and antigranzyme K–PE (Santa Cruz Biotechnology). Isotype control was used for every staining using the isotypes of antibodies included in the staining panel. The cells were examined with FACScan type flow cytometer (BD Bioscience). Data analysis was performed using FlowJo Software (Tree Star). The analysis was performed on events characterized by the medium size (FSCmed) and low granularity (SSClow).

The obtained data were subjected to statistical analysis. The consistency of quantitative variables with normal distribution was analyzed with the Shapiro–Wilk and Kolmogorov–Smirnov test. The homogeneity of variance within the groups was tested with the Levene test. Based on the verification of these assumptions, the variables were analyzed with the nonparametric Mann–Whitney *U* test. The relationship between analyzed variables and patient's age was tested with the simple Pearson's coefficients of correlation (*r*), also after identification of outliers with the Grubbs’ test and their elimination. The significance of all statistical tests was set at commonly used levels (ie, *P* < 0.05, *P* < 0.01, and *P* < 0.001). All calculations were conducted with Statistica 9.0 package (StatSoft, Krakow, Poland).

The protocol of the study was approved by the Local Bioethical Committee of Ludwik Rydygier Collegium Medicum, Nicolaus Copernicus University in Torun.

## RESULTS

**FIGURE 3:**
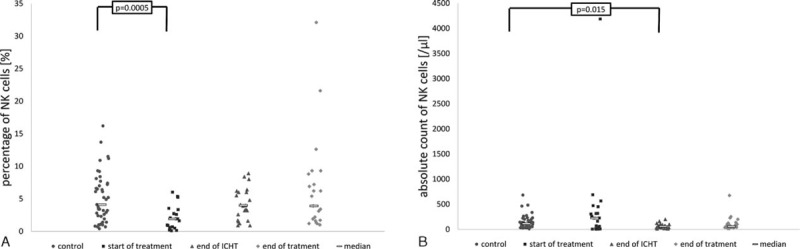
Percentage and absolute count of NK cells in the studied groups and in the controls. At start of treatment n = 21 patients, at the end of ICHT n = 22 patients, at the end of treatment n = 23 patients, controls n = 43 patients. ICHT = intensive chemotherapy, NK = natural killer.

Percentage and absolute number of NK cells in peripheral blood. The most pronounced abnormalities in the percentage of NK cells were observed in group I (ie, at diagnosis of neoplastic disease). The percentage ranged from 0.1% to 6% (Me 1.9%) and was significantly lower than in the controls (range 0.4–16.2%, Me 4.1%; *P* = 0.0005). The percentages of NKs in groups II and III ranged between 0.9% and 8.9% (Me 4%) and between 1% and 32.1% (Me 3.9%), respectively, and did not differ significantly as compared with the controls (Fig. 3A).

The absolute number of peripheral blood NKs at diagnosis of malignancy was highly variable (from 4 to 4188 per μL, Me 223 per μL). The counts were markedly lower after completing both the intense phase of chemotherapy (11–200 per μL, Me 56.5 per μL) and the entire oncological treatment (14–675 per μL, Me 56 per μL). Compared with the controls (range 11–682 cells per μL, Me 111 per μL), statistically significant difference was documented solely in group II (*P* = 0.015; Fig. 3B).

### Expression of Intracellular IFN-γ

**FIGURE 4:**
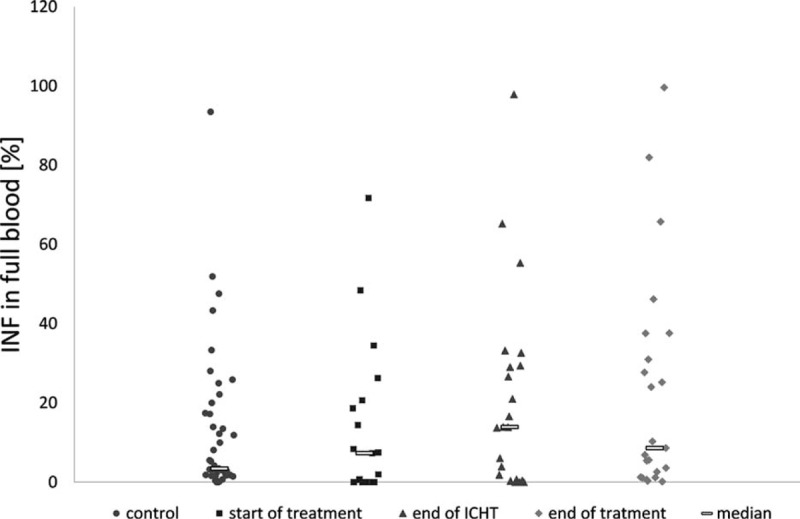
Expression of intracellular INFγ in NK of the studied groups and the controls. At start of treatment n = 21 patients, at the end of ICHT n = 22 patients, at the end of treatment n = 23 patients, controls n = 43 patients. ICHT = intensive chemotherapy, INFγ = interferon-γ, NK = natural killer.

Both studied groups and the controls were characterized by highly variable fraction of cells showing the expression of IFN-γ. At each stage of the therapy, the median of patient's positive cells was higher than that of the controls. The percentage of IFN-γ-positive NKs ranged from 0% to 71.7% in group I (Me 7.3%), from 0% to 97.8% (Me 13.9%) in group II, and from 0.2% to 99.6% (Me 8.6%) in group III. In the controls, the percentage of cells showing the intracellular expression of IFN-γ varied from 0% to 93.4% (Me 3.4%). These differences did not prove significant at any stage (Fig. 4).

### Expression of CD158a and CD158b on the Surface of NKs

**FIGURE 5:**
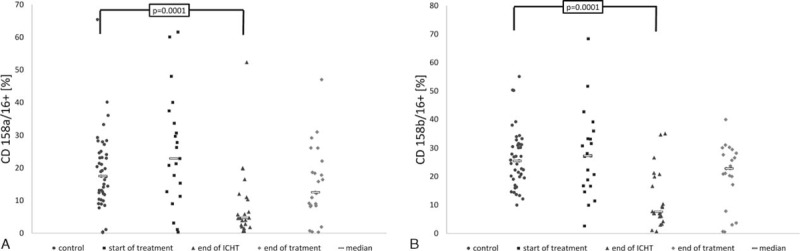
Expression of CD158a/CD16+ (A) and CD158b/CD16+ (B) on the surface of NKs in studied groups and the controls. At start of treatment n = 21 patients, at the end of ICHT n = 22 patients, at the end of treatment n = 23 patients, controls n = 43 patients. ICHT = intensive chemotherapy, NK = natural killer.

The highest, but still not significantly different, values were documented at diagnosis of the malignancy (CD158a: 0.3–61.6%, Me 22.9%; CD158b: 2.6–68.4%, Me 27.3%). In this case, the most pronounced defects were observed after completing the intense phase of the chemotherapy. At this time, the fraction of NK cells with the expression of both analyzed markers was the lowest, ranging from 0.6% to 52.3% (Me 4.7%) for CD158a, and from 0.7% to 35.1% (Me 7.6%) for CD158b. These values proved to be significantly different as compared with the controls (CD158a: 0.2–65.4%, Me 17.5%, *P* = 0.0001; CD158b: 10.0–55.1%, Me 25.5%, *P* = 0.0001). After completing oncological therapy, the expression of both CD158a (0.3–47.0%, Me 12.5%) and CD158b (0.5–39.9%, Me 20.7%) was only slightly lower than in the controls (Fig. 5A and B).

### Expression of Perforin and Granzymes A, B, and K

**FIGURE 6:**
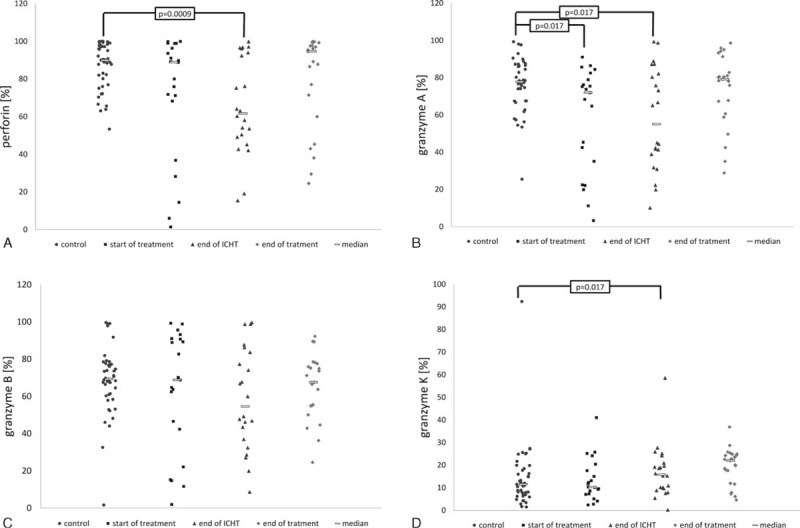
Expression of perforin (A), granzyme A (B), granzyme B (C), and granzyme K (D) in NKs in studied groups and the controls. At start of treatment n = 21 patients, at the end of ICHT n = 22 patients, at the end of treatment n = 23 patients, controls n = 43 patients. ICHT = intensive chemotherapy, NK = natural killer.

The percentage of NKs with the expression of perforin in children who completed the intense phase of chemotherapy (15.9–99.8%, Me 61.7%) was markedly lower than in the healthy subjects (53.0–100%, Me 89.9%; *P* = 0.0009). The differences in the remaining groups proved insignificant; at diagnosis, perforin was detected in 1.4% to 100% (Me 88.8%) of NKs, while at the end of the treatment this marker was found in 24.5% to 99.8% (Me 94.6%) of the cells (Fig. 6A).

In group I, the percentage of NKs with the expression of granzyme A ranged between 3.3% and 91.0% (Me 71.9%). Similar levels of expression were documented in patients who completed the intense phase of the chemotherapy (range 10.1–99.3%, Me 55.1%). These values were significantly lower compared with the controls, in whom granzyme A was detected in 25.4% to 99.3% (Me 77.7%) of the cells (*P* = 0.017 for both groups). The percentage of positive cells in children who completed the treatment was similar as in the healthy individuals (range 28.8–98.6%, Me 79.1%; Fig. 6B).

The percentage of NKs showing the expression of granzyme B was the lowest in children after the intense phase of the chemotherapy (range 8.7–99.6%, Me 54.6%), but the difference did not prove significant in compared with the controls (1.6–99.6%, Me 69.3%). The level of expression in the remaining groups was similar as in the controls and amounted to 1.6% to 99.3% (Me 68.8%) at diagnosis, and 24.5% to 92.2% (Me 67.5%) at the end of the treatment (Fig. 6C).

The fraction of NK cells showing the expression of granzyme K in group III was significantly higher than in the controls (range 4.6–36.8% vs 1.5–92.3%; Me 22.0% vs 11.5%; *P* = 0.005). The percentage of NKs expressing granzyme K in groups I and II patients ranged from 2.4% to 41.2% (10.3%) and from 0.2% to 58.5% (Me 15.8%), respectively; these values did not prove significantly different as compared with the controls (Fig. 6D).

**FIGURE 7:**
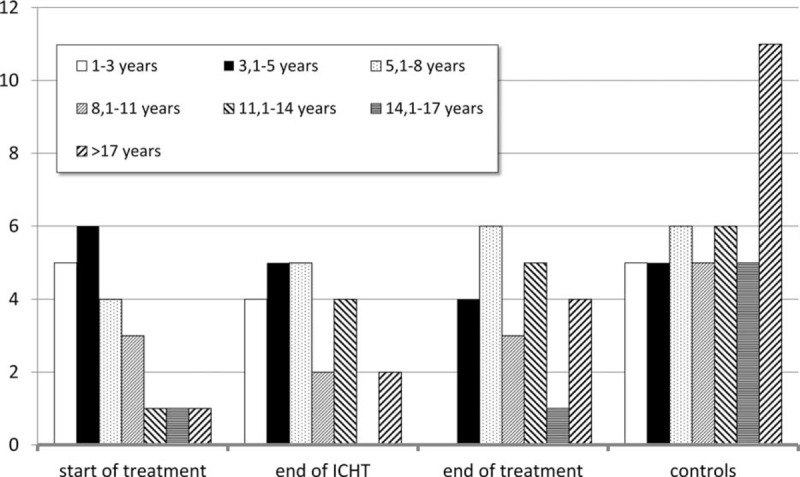
Distribution of age in studied groups and in the controls.

Due to the high variability of age in the studied group and healthy controls (range 13–22 years), the relationship between patient's age and the values of studied parameters was verified. Noticeably, group I was predominated by younger children, between 1 and 8 years of age, whereas the control group comprised the highest fraction of individuals older than 17 years (Fig. 7).

Analysis of the simple coefficient of correlation with age did not confirm the relationship of particular variables both in the whole group and in the controls. Analysis within particular studied groups confirmed the relationship between patient's age and the expression of perforin (*r* = −0.4337, *P* = 0.05) at diagnosis of malignancy, as well as the expression of CD158a (*r* = −0.4176, *P* = 0.047), granzyme A (*r* = −0.4326, *P* = 0.039), and granzyme K (*r* = −0.4166, *P* = 0.048) at the end of oncological therapy (Table 2). After excluding outliers, the percentage of NKs in full blood proved to be the only parameter associated with patient's age in the whole population of both diseased and healthy individuals (*r* = 0.228, *P* = 0.019 and *r* = 0.3246, *P* = 0.038, respectively). Analysis after elimination of outliers with the Grubbs’ test revealed a significant relationship between patient's age and the expression of CD158b (*r* = −0.4697, *P* = 0.037) and perforin (*r* = −0.544, *P* = 0.016) at diagnosis, as well as between the age and the expression of CD158a (*r* = −0.4176, *P* = 0.047), granzyme A (*r* = −0.4166, *P* = 0.048), and granzyme K (*r* = −0.4166, *P* = 0.048) after completing the therapy (Table 3).

**TABLE 2:**
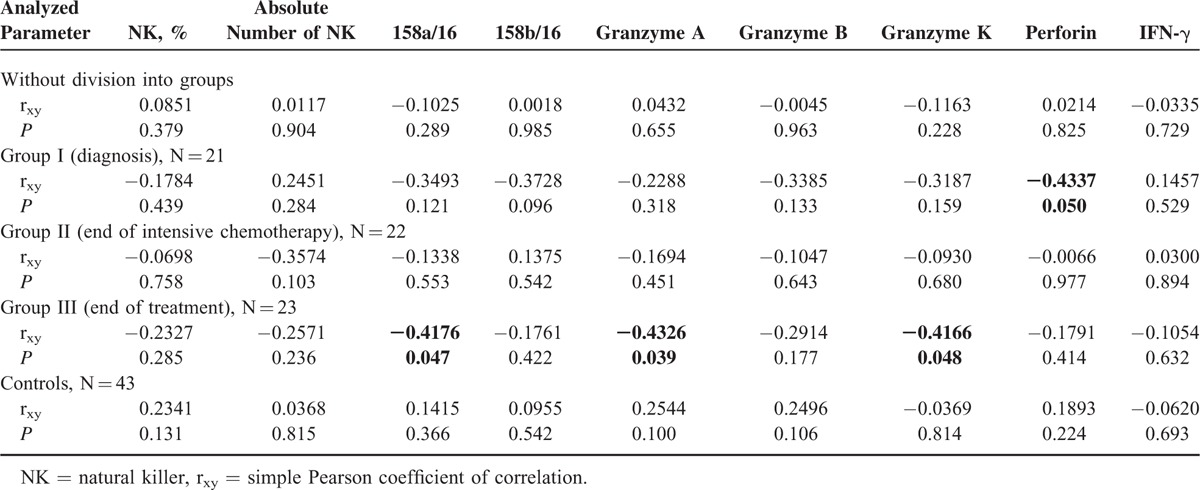
Simple Pearson Coefficients of Correlation Between Analyzed Variables and Age at Examination

**TABLE 3:**
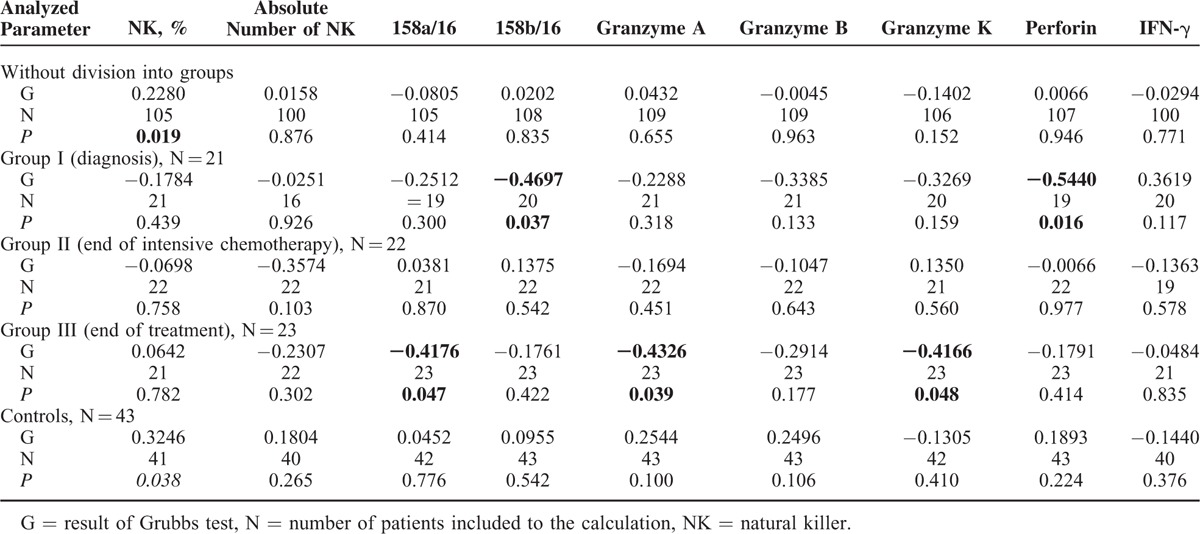
Coefficients of Correlation Between Analyzed Parameters and Age at Examination After Identification of Outliers

## DISCUSSION

Development of methods enabling rapid and reliable detection of NK cells, followed in the future by the development of cytotoxicity tests, and assessing their functional capacity, has constituted the base of studies addressing the role of these cells in various conditions. The research on the complex mechanisms employed by NKs to execute their biological functions was initiated considerably later.

In this study, we analyzed the functional potential of NKs by means of determining their phenotype on the basis of selected immunological parameters. Obtained data enabled us to understand the mechanisms underlying the cytotoxicity-related disorders of these cells. Analysis of the expression of KIR surface receptors (CD158a and CD158b), perforin, and the intracellular expressions of A, B, and K granzymes involved NK cells that were isolated by negative selection method, with an aid of SuperMACS separator. As early as in 2005, Leung et al^19^ revealed that this procedure does not modulate the expression of their surface receptors or the intracellular expression of cytokines, perforin, and granzyme B.

### Analysis of Percentage and Absolute Count of NKs in Peripheral Blood

The analysis by Dębski et al,^20^ involving 58 children at diagnosis of ALL, revealed a decrease in the percentage of NKs along with slightly less evident decrease in their absolute count. At the same stage, Węclawek-Tompol et al^21^ observed that the absolute number of NKs was higher than in the controls. Our study revealed a significant decrease in the percentage of these cells (*P* < 0.001), but without corresponding significant decrease in their absolute count. It must be noted, however, that the findings reported in adult patients were contradictory.^22^ After completing intense chemotherapy by our patients, the absolute count was the only significantly lower NK parameter; we interpreted this finding in terms of post-therapeutic leucopenia, which is typical for this stage. This phenomenon was previously documented by other authors.^21–23^ In contrast, Jarosz et al^24^ revealed that children at this stage of therapy are characterized by significantly decreased percentage and absolute count of NKs.

Prior studies dealing with the problem in question involved patients who completed the complex therapy of ALL; however, the period between the withdrawal of the therapy and NK count determination varied between 1 day and more than 5 years.^23,25,26^ Our study focused on the analysis of the effects of many-week ALL remission maintenance therapy on NKs; therefore, we analyzed the percentage and absolute count of these cells 2 weeks after completing the treatment. Following this period, the direct reducing effect of cytostatics on peripheral blood leukocytes, including lymphocytes, resolves. No significant differences, both in percentages and absolute counts, were documented when the patients were compared with the controls. Another study, conducted on the day of therapy withdrawal, revealed that both the percentage and absolute count of NKs were reduced; in contrast, both these parameters were similar as in healthy controls as early as 1 month later.^26^

No relationship between patient's age and the percentage/absolute count of NKs was documented both in the patients and in the controls. The percentage of NKs was the only parameter showing such a relationship after removing the outliers. The values were markedly lower in the group of 1- to 3-year-old children. The same tendency was previously documented by Sundström et al.^27^ In contrast, Piątosa et al^28^ did not reveal an association between the age and percentage/absolute count of NKs, except during the first few days of life when both these parameters were markedly higher. Similarly, no gender- and age-related variability in the percentage and absolute count of NKs was observed in children between 3 and 19 years of age.^29^

### Expression of Intracellular IFN-γ in NK Cells

About 5% of NKs present in peripheral blood show the expression of CD56 bright and execute their regulatory and effector function via the release of cytokines, including IFN-γ.^30^ The principal role of this cytokine pertains to the control of viral infection as well as to the elimination of cells that have undergone neoplastic transformation.^31^ Since NK expression of interferon can be undetectable at rest, it is most commonly determined following the stimulation (eg, with IL-2, IL-12, or lipopolysaccharides).^10,32^ Moreover, nitric oxide involved in the lipopolysaccharides dependent activation of inducible nitric oxide synthase (NOS-2) is secondary to the endothelial nitric oxide synthase (NOS-3) activation. These processes are induced by local interaction between inflammatory process cells and endothelium. Nitric oxide acts predominantly as a regulator of muscle tension in the local system: endothelium—the muscular vessel. It is also a factor inhibiting angiogenesis and adhesion, aggregation of platelets. The first study suggesting the role of NOS-3 in generation of NO-related hyporeactivity during early sepsis was presented in 2001.^33,34^

In our study, the expression of IFN-γ by NKs was analyzed without stimulation, and no significant intergroup differences were documented. One could suppose that at diagnosis of ALL the neoplastic cells would represent a trigger factor of NK activation, and therefore the expression of IFN-γ should be higher than in healthy individuals. However, these assumptions were not confirmed by our findings, suggesting that the presence of lymphoblasts is insufficient for NK activation and induction of their effector function. Also, previous studies analyzing the expression IFN-γ upon stimulation in ALL patients at diagnosis or recurrence, as well as during the maintenance therapy or upon its withdrawal, revealed a markedly lower expression of this cytokine as compared with healthy individuals.^22,35^

### Expression of CD 158a and CD158b on the Surface of Isolated NKs

According to the “missing self” hypothesis, the transmission of activating signal and/or the simultaneous weakening of cell-inhibiting stimuli is the first necessary component of NK activation.^8,9^ This initial, critical component results from the interaction between activating and inhibiting receptors of NKs and their ligands present on target cells.^14–16,36^ KIRs play an important role among NK receptors. They include both inhibiting and activating KIRs. The set of KIRs in a given individual is determined genetically. However, not all receptors encoded in genome are represented on every NK clone.^37,38^

In our study, we analyzed the expression of CD158a molecule, which corresponds to 2DL1 inhibiting receptor and 2DS1 activating receptor, and CD158b, identifying inhibiting receptors 2DL2 and 2DL3. The dual specificity of monoclonal antibodies resulted from extremely high structural homology of the above-mentioned pairs of receptors.^39^

Our study revealed the presence of 2DL1 gene in nearly all individuals from the studied and control group (98% and 100%, respectively), whereas the 2DS1 gene was present in more than 1/2 of the patients and in <40% of the healthy subjects (not shown). The percentage of NKs from healthy individuals showing overexpression of CD158a was 17.5%. This value closely resembles the result of population-based study performed in Brazil.^40^ The results of our patients differed depending on analyzed time point. At diagnosis of ALL, the percentage of NK showing CD158a expression was nonsignificantly higher; this parameter was the lowest in group II (after intense chemotherapy; *P* = 0.0001), and only slightly decreased after completing the treatment.

Genes 2DL2 and 2DL3 were detected in all studied individuals and controls (not shown). In turn, the median percentage of NKs showing the presence of 1 or 2 receptors identified by anti-CD158b antibodies in healthy individuals was 25.5%. Similar results (Me 26.4%) were previously documented in Brazilian population.^40^ In our study, the fraction of NKs showing the expression of CD158b at the end of intense chemotherapy was significantly lower than in the controls (*P* = 0.0001).

Feuchtinger et al analyzed the sensitivity of lymphoblasts derived from line B precursors of 285 various NK clones and revealed that elements determining their reactivity included the expression of 3 principal inhibiting KIRs: CD158a, b, and e (ie, 2DL1, 2DL2/2DL3, and 3DL1) and the presence of ligands for particular receptors on neoplastic cells.^39^ The presence of at least 1 inhibiting receptor KIR on NKs and its corresponding ligand on lymphoblasts was reflected by a significant decrease in the sensitivity of neoplastic cells.^41^ In this study, we did not analyze the presence of class I histocompatibility antigens in our patients nor the expression of these antigens on lymphoblasts. Nevertheless, patients at diagnosis of ALL were the only group in which the percentage of NKs showing the expression of both CD158a and CD 158b was higher than in the controls.

Our study revealed that multi-agent intense oncological therapy represents a factor that evidently disturbs the NK expression of CD158a and CD158b. Compared with healthy individuals, patients who completed intense chemotherapy showed extremely low percentage of cells showing the expression of both receptors (*P* = 0.0001). This suggests that the defect of KIR expression, and probably also the impaired expression of other receptors, increases proportionally to the intensity of the treatment. Leung et al studied 14 survivors after at least 18 months from completing the therapy of ALL. They observed that the absolute number of peripheral NK cells showing the expression of KIR2DL1 (ie, CD158a) and KIR2DL2/3 (CD158b) was lower than in the case of NK cells obtained from healthy siblings of studied patients.^42^ This suggests that the disordered expression of analyzed KIRs not only occurs during the treatment (as shown in our study) but persists markedly longer. The authors of the above-mentioned study proposed that the impaired cytotoxicity associated with a lower expression of these receptors can represent the risk factor of secondary malignancies.^42^ In studies presented by Mi et al,^43^ in patients with MDS and AML, CD158a expression was significantly lower than in the control group, whereas there was no difference in the CD158b expression.

Our analysis did not document a relationship between the expression of CD158a and CD158b on the surface of NKs and patient's age, both in the whole studied group and in the controls. Also, Almeida-Oliveira et al^40^ did not reveal an association with age of healthy individuals. In some studies, Lutz et al^44^ and Manser et al^45^ suggested that the expression of KIRs increases with age. In contrast, the comparative study of KIR expression in umbilical (representing the principal source of stem cells for transplantation) and peripheral blood revealed that this earlier material contained slightly higher percentage of NKs expressing CD158a and a significantly lower fraction of these cells showing the expression of CD158b.^46^ The fact that the percentage of CD158a- and CD158b-positive cells in both healthy and diseased 12-month-old children participating in our study was similar to that in older individuals suggests that these parameters are stabilized as early as during the first year of age. The analysis of specific groups of patients confirmed the association between the expression of CD158a and the age of patients who completed oncological therapy, both in simple correlation test and after elimination of outliers with the Grubbs’ test. The most pronounced disorders were observed in older children. Similar association with age was revealed in the case of CD158b expression determined at diagnosis of ALL.

### Expression of Perforin and A, B, and K Granzymes Within NK Cells

Synthesis and release of proteases, namely perforin and granzymes, represents 1 of the most important mechanisms by which NK cells execute their effector function associated with direct cytotoxicity.^14–16^ Perforin is responsible for making the holes in cellular membrane through which granzymes enter the target cell and cause its death by inducing apoptosis. The cytotoxicity of NKs seems proportional to their content of perforin and granzymes.^47–49^

#### Perforin

Our study showed reduced expression of perforin solely in NKs of patients who completed intense chemotherapy. This suggests that this phenomenon results mostly from the influence of environmental factors; most likely, the therapeutic agents. Voskoboinik et al focused on severe perforin deficiencies, which occur in patients with familial lymphohistiocytosis. Furthermore, they pointed to the possible risk of more frequent development of leukemia in patients with partial defect of gene determining the synthesis of perforin.^49^ In patients with MDS and AML percentage of NK, expressing perforin was also lower than in the control group.^43^

We did not observe any age-specific variability in perforin expression, either in the whole studied population or in the controls. Nevertheless, there was an inverse correlation at diagnosis of ALL, suggesting that older children had the lowest expression of perforin. The lack of a relationship between perforin expression and the age of healthy individuals was confirmed by Wang et al.^46^ The expression of intracellular perforin determined in umbilical blood did not differ significantly from the analogous parameter analyzed in the peripheral blood of adult individuals.^46^

#### Granzyme A

Granzyme A, a representative of serine proteases, shows enzymatic activity which is comparable to trypsin and is a crucial enzyme present in the granules of cytotoxic NKs. Although the mechanisms by which granzyme A leads to the death of target cell vary, the degradation of single DNA threads seems to play the most important role. The stimulation of proinflammatory cytokines constitutes its additional function.^47,48,50^

In our study, the expression of intracellular granzyme A was significantly lower in patients at diagnosis of ALL and after completing intense chemotherapy than in healthy individuals. Sparse available studies revealed that induction of apoptosis by perforin/granzyme system is the most important mechanism used by NKs for efficient killing of lymphoblasts.^51,52^ The reduced expression of granzyme A can be considered as 1 of the ways that lymphoblasts can “escape” from the immunological control executed by NKs. Other analyses confirmed that induction of apoptosis by granzyme A and caspase 3 is 1 of the principal mechanisms underlying the effect of glucocorticoids in the therapy of ALL.^53–55^ Consequently, it can be concluded that the efficacy of these agents used in prophase and induction is associated with the correction of the relevant NK defect. Dexamethasone is used during the final phase of intense chemotherapy, which should provide normal synthesis of granzyme A during this stage of treatment. However, this assumption was not confirmed by the findings of our study since the expression of intracellular granzyme A in group II subjects was significantly lower. Therefore, it is more reasonable to assume that the expression of granzyme A during this stage is determined by the action of various therapeutic agents, both stimulating and inhibiting the synthesis of this molecule by NKs.

The simple coefficient of correlation did not reveal a relationship between the expression of granzyme A and the age of studied and control subjects. However, the relationship between this analyzed parameter and patient's age was observed at the end of oncological therapy. The values were the lowest in older children.

#### Granzyme B

Granzyme B, a serine protease present in the granule of cytotoxic NKs, shows enzymatic activity similar to that of chymotrypsin. It was proved that although the effector cells that lack granzyme B are capable of killing target cells, their efficacy is markedly lower.^42,44^ They act through the activation of caspase 3 and 7, via an indirect or direct mechanism. Caspases cause degradation of many cellular proteins, as a result leading to programmed cell death.^44,46^

Our study did not reveal any disorders of granzyme B at any stage of the disease. This suggests that the synthesis of this protease is not markedly modulated by environmental factors, and that it can be responsible for relatively efficient functioning of NKs after achieving remission, but still during the treatment, as it was postulated by many authors.^22,56,57^ In contrast, it has been reported in patients with MDS and AML significantly lower percentage of NK expression of granzyme B in relation to the control group.^43^ Perhaps this is due to a different tumor biology. Results of some recent studies suggest that it may be secondary to the different ability of cancer cells to produce a specific protease inhibitor 9, inhibiting the activity of granzyme B.^58^

The association between granzyme B expression and patient's age has not been confirmed by any experimental study. The only published study analyzing the content of granzyme B in umbilical blood revealed significantly lower expression of this molecule as compared to adult individuals.^42^ Probably, the expression of granzyme B in 12-month-old children (the youngest individuals from the studied and control group) is similar to that observed in adults.

#### Granzyme K

Granzyme K shows trypsin-like enzymatic activity. It can induce rapid death of cells with a single DNA thread irrespective of caspase activation, through the induction of endoplasmic reticulum associated complex, or induction of production of reactive oxygen species.^59–61^ Transcription factor p53, which plays the role of neoplastic cell suppressor, is a physiological substrate of granzyme K. The products of “cutting” p53 by granzyme K show strong pro-apoptotic properties for neoplastic cells.^60,62^ The “escape” of neo-plastic cells from the destruction executed by this mechanism is possible due to the mutation of p53-encoding gene, observed in approximately 50% of human malignancies, or the impairment of its function, documented in about 80% of the tumors.^61,63^ Both murine and human model of leukemia, including B-line leukemia, revealed the functional disorders of p53.^63,64^ Our study revealed that patients who completed therapy (group III) were characterized by the elevated expression of granzyme K. Analyses of granzyme K expression during other conditions showed that it is level is increased in sepsis and respiratory pathologies.^46,65,66^ In sepsis, serum content of soluble granzyme K was markedly elevated, while during the septic shock it was significantly decreased as compared with healthy adults. The elevated concentration of granzyme K was explained by lower expression of inter-alpha inhibitory protein (IaIp), which is considered to be its natural neutralizer.^65^ Several studies have confirmed the impaired regulation of the synthesis of serine protease inhibitor. Lower expression of the inter-alpha inhibitory protein was observed in breast, colon, lung, and prostate cancer,^67^ while an elevated expression was documented in gastric cancer patients.^68^ However, there is no similar, published research regarding ALL. Moreover, it is unclear if IaIp can modulate the expression of intracellular granzyme K (ie, the parameter), which was measured in patients and controls taking part in our study, instead of the serum level of free granzyme K.

Both analysis of simple correlation and the analysis after elimination of outliers with the Grubbs’ test did not confirm the relationship between granzyme K and the age of the whole studied group and the controls. However, the inverse correlation of this parameter was observed in both tests in patients who have completed the treatment, suggesting that its values were the lowest in older children.

## CONCLUSIONS

Although the impaired number and percentage of NKs showing the intracellular expression of INFγ, perforin, and granzymes A, B, and K was documented at every analyzed stage, the abnormalities were most pronounced after completing intense chemotherapy, and least evident after the maintenance therapy of remission in ALL.

Significant decrease in the percentage of NKs and the content of these cells showing the intracellular expression of granzyme A was revealed at diagnosis of ALL; however, these abnormalities seem to reflect the effect of malignancy on the function of studied cells rather that their role in ALL pathogenesis.

The disorders of NK phenotype documented after completing intense chemotherapy seem to be induced by administered agents, suggesting that environmental conditions play an important role in the modulation of genetically determined traits. Significant differences observed at this stage of ALL treatment pertained to many parameters characterizing the functional capacity of cells: lower absolute number of NKs, lower percentage of cells showing surface expression of KIR (CD158a and CD158b), as well as a lower fraction of NKs with intracellular expression of perforin and granzyme A.

After completing the maintenance therapy of remission in ALL, the only significant difference pertained to the higher percentage of NKs with intracellular expression of granzyme K. Probably, higher expression of this molecule reflects the activation of compensatory mechanisms of the body, which are aimed at improving the functional capacity of NK cells.
